# An updated meta-analysis of cardiac resynchronization therapy with or without defibrillation in patients with nonischemic cardiomyopathy

**DOI:** 10.3389/fcvm.2023.1078570

**Published:** 2023-07-12

**Authors:** Fuwei Liu, Xin Gao, Jun Luo

**Affiliations:** Department of Cardiology, Ganzhou People’s Hospital, Ganzhou, China

**Keywords:** nonischemic cardiomyopathy, heart failure, cardiac resynchronization therapy, CRT defibrillators, CRT pacemaker, mortality

## Abstract

**Background:**

Cardiac resynchronization therapy (CRT) is a major device therapy used to treat patients suffering from heart failure (HF) and electrical asynchrony. It can improve HF symptoms, reduce HF hospitalization time, and improve long-term survival in HF with and without implantable cardioverter (ICD) therapy. However, the benefit of defibrillator therapy in CRT-eligible patients with nonischemic cardiomyopathy (NICM) remains unknown. As a result, we conducted a systematic review and meta-analysis to compare clinical outcomes in patients with NICM and HF who were treated with implantable CRT defibrillators (CRT-D) vs. a CRT pacemaker (CRT-P) alone.

**Methods:**

We searched the electronic databases PubMed, Embase, and Cochrane for all studies comparing CRT-D vs. CRT-P treatment in patients with NICM. The time frame was from 1990 to September 2022. All-cause mortality and cardiovascular mortality were the primary clinical outcomes of interest to us. To pool adjusted hazard ratios (HRs) and 95% confidence intervals (CIs), a random-effects model with inverse variance was used.

**Results:**

A pooled meta-analysis included two randomized controlled trials (RCTs), each with 1,200 CRT-eligible patients with NICM (592 with CRT-D and 608 with CRT-P) and nine cohort studies representing 27,568 CRT-eligible patients with NICM (16,196 with CRT-D and 11,372 with CRT-P). The adjusted HR for all-cause mortality for CRT-D vs. CRT-P was 0.90 (95% CI, 0.81-0.99). In a subgroup analysis of two RCTs and nine cohort studies, the adjusted HR for all-cause mortality was 0.72 (95% CI, 0.43–1.19) and HR 0.92 (95% CI, 0.83–1.03) for CRT-D vs. CRT-P, respectively.

**Conclusion:**

With the addition of defibrillation leads, we found a significantly lower risk of all-cause mortality in patients with NICM, but this association was not found in subgroup analyses of RCTs and observational studies.

## Introduction

Heart failure (HF) is a leading cause of morbidity and mortality worldwide and one of the most common cardiovascular diseases in the United States. According to one survey, 6.2 million Americans suffer from HF, and $30.7 billion is spent on HF treatment each year (1). Over the last two decades, advances in HF drug therapy have reduced the risk profile of patients with reduced ejection fraction (HFrEF) by 44%, as has the risk of sudden cardiac death (SCD) (2). The advent of device therapy for HF has transformed the long-term prognosis of HF patients.

In the management of HF, cardiac resynchronization therapy (CRT) represents a significant advancement. By resynchronizing intraventricular conduction/contraction and encouraging favorable reverse remodeling, CRT increases the quality of life and survival in patients with ventricular asynchrony and reduced left ventricular ejection fraction. Its efficacy has been proven in numerous randomized controlled trials (RCTs) (3–5). Patients who require pacing and have a left ventricular ejection fraction (LVEF) of 36% to 50%, as well as those with HF who are older or have many comorbid conditions, typically use CRT pacemakers (CRT-P). Implantable cardioverter-defibrillators (ICDs) are currently recommended for patients with HF, EF ≤ 35%, or who continue to be symptomatic despite receiving optimal drug therapy. However, in patients with HFrEF who are qualified for both primary SCD prevention strategies and indications for CRT, the need for Implantable cardioverter-defibrillators (ICD) implantation is frequently controversial. Indeed, many patients are candidates for both CRT and ICDs. CRT-defibrillators (CRT-D) are now recommended for patients with nonischemic cardiomyopathy (NICM) and ischemic cardiomyopathy (ICM) who have a long QRS duration and LVEF ≤ 35% (6).

There is limited availability of recent, randomized data that directly evaluate the efficacy of ICD in CRT patients, i.e., a CRT-D vs. CRT-P comparison. The DANISH trial, which was completed in 2016, revealed that ICDs may not be beneficial for patients with NICM (7). Previous meta-analyses have raised concerns about this result (8–11). Notably, Golwala et al. used a pooled cohort and demonstrated a 23% reduction in all-cause mortality in NICM patients treated with an ICD. However, because the data analyzed only included the CRT-D and optimal drug therapy (OPT) groups, not the CRT-D vs. CRT-P group, these studies were unable to precisely quantify the effect of ICD use on patients with implanted CRT (8, 10). The latest systematic review and meta-analysis included a randomized controlled trial with a subgroup of 645 CRT-eligible NICM patients (322 CRT-D and 323 CRT-P) and 7 observational studies with 9,944 CRT-eligible NICM patients (6,865 CRT-D implantations and 3,079 CRT-P implantations). The findings revealed no link between increased defibrillator therapy and lower all-cause mortality in CRT-eligible NICM patients (12). Given the limited number of clinical studies comparing CRT-D to CRT-P in the NICM population, the lack of clear benefits suggested by previous meta-analyses and the controversy raised by ongoing published clinical studies. Therefore, it is necessary to re-evaluate the impact of using CRT-D vs. CRT-P on the clinical outcomes of NICM patients.

## Methods

The Preferred Reporting Items for Systematic Reviews and Meta-Analyses statement was followed in our research (13).

### Search strategy

We searched the PubMed, EMBASE, and Cochrane databases from 1990 to September 2022 to identify studies evaluating CRT with primary prevention in patients with NICM. We designed the search strategy ahead of time based on the differences in the database. The following two subject terms will be combined with the Boolean operator “and” in our search strategy: (1) cardiomyopathies OR cardiomyopathy. (2) cardiac resynchronization therapy OR cardiac resynchronization therapy OR CRT. We did not restrict the literature search by language. The list of references from the included studies was screened to find additional pertinent studies, preventing literature omission.

### Eligibility criteria

The following were the inclusion requirements for this study: (1) patients who were selected with NICM diagnoses; (2) CRT-D vs. CRT-P; and (3) outcomes: all-cause mortality, cardiac cause of mortality, hospitalization. Any associated procedural and postprocedural risks were also of interest. (4) Effect estimates: adjusted hazard ratios (HRs) and 95% confidence intervals (CIs). Reviews, case series, case reports, letters, editorials, guidelines, and conference abstracts were all excluded because they did not contain effect estimates. The study with the largest sample size or the longest duration was chosen as the final inclusion study if patients from different studies came from the same data source.

### Data extraction and quality appraisal

Two investigators (*FW-Liu* and *X-Gao*) performed all study screens. The first author’s name, the publication journal, the date of publication, the type of study, the database source, the sample size, the primary outcome endpoint, any secondary outcomes, and the model corrected for confounders were all taken from the included studies. Any disagreements that arose during the process were left to a third investigator (*J-Luo*) who made the final decision on inclusion in the meta-analysis. The authors of any studies with insufficient data to be included in the meta-analysis were contacted twice to obtain the information they needed. The study would not be taken into account for the quantitative analysis of the species if there was no response or insufficient data, or it would only be taken into account for the qualitative analysis.

### Risk of bias assessment

The Cochrane risk of bias assessment tool assessed the methodological quality of RCTs as well as *post hoc* RCT analysis. For observational cohort studies, the Newcastle‒Ottawa Scale (NOS) tool was used to appraise study quality. The NOS tool had three major sections: cohort selection (0–4 points), cohort comparability (0–2 points), and outcome assessment (0–3 points). We regarded an NOS score of ≥6 points as moderate-to-high quality and an NOS score of <6 points as low quality (14).

### Data synthesis and statistical analysis

In Review Manager 5.4, outcomes were quantitatively pooled when appropriate (the Nordic Cochrane Center, Rigshospitalet, Denmark). The random effects model was used to calculate adjusted HRs for clinical outcomes with 95% CIs. The Cochrane statistic I^2^ was used to assess study heterogeneity. An I^2^ threshold value of ≥60% indicates significant heterogeneity between studies, which needs to be explored by the researcher.

## Results

### Article extraction

**Figure 1:**
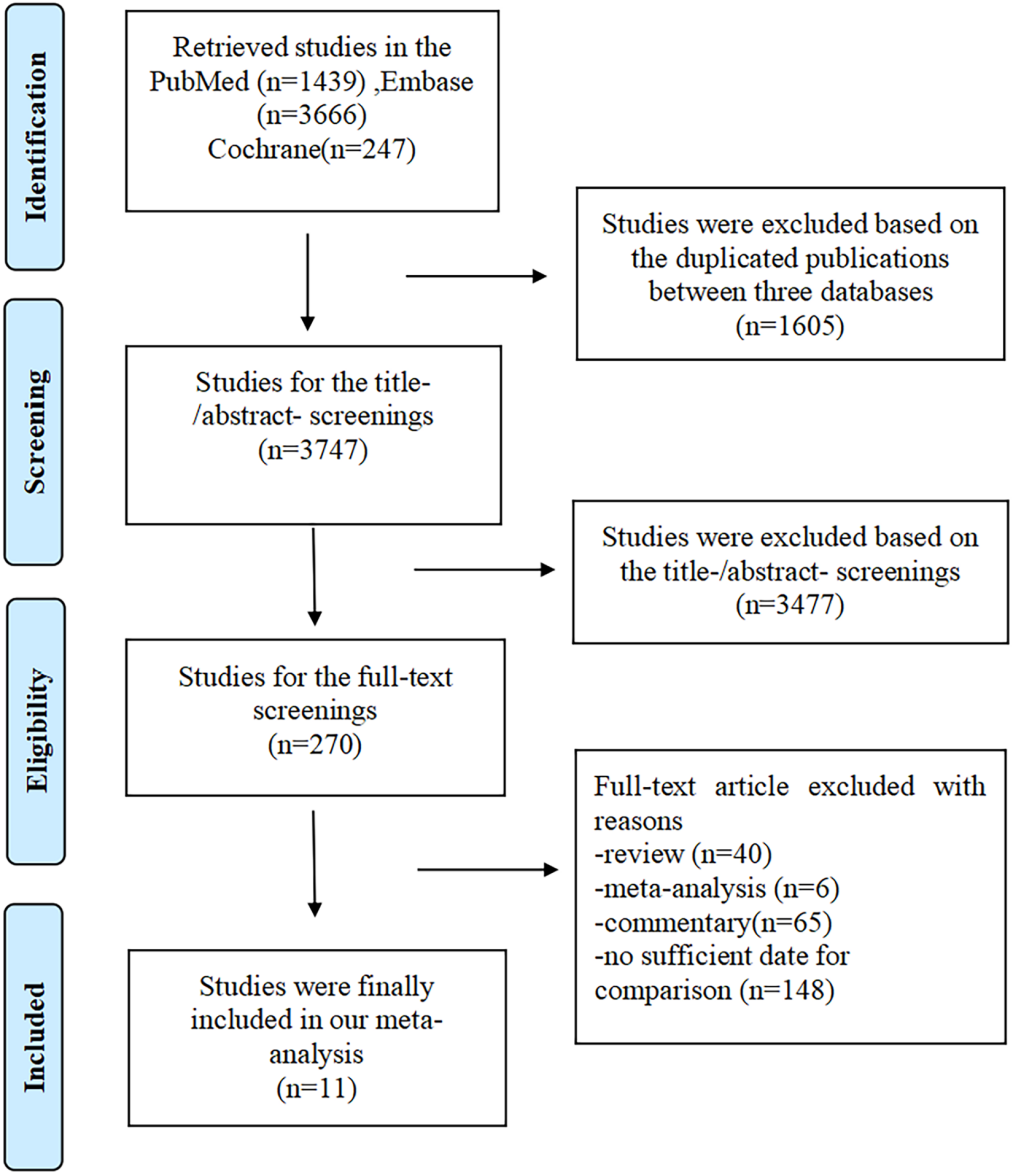
The process of the literature retrieval of this meta-analysis.

Using a predefined search strategy, we extracted 5,352 studies from the database (Supplementary Table S1 in the Data Supplement). By removing duplicates, we were able to exclude 1,605 studies. We excluded 3,477 irrelevant studies by reviewing the titles, abstracts, and keywords based on the predefined inclusion and exclusion criteria. The full text of 270 citations was thoroughly reviewed by the investigators. We excluded 40 reviews, 6 meta-analyses, 65 reviews, and 148 studies that did not include a CRT-D vs. CRT-P control. The quantitative analysis eventually included a total of 11 studies (Figure 1).

### Study characteristics and risk of bias

Finally, we included 11 studies, 2 of which were RCTs (7, 15) and 9 of which were cohort studies (16–24). There were 11 studies that reported on all-cause mortality and 3 studies that reported on cardiac mortality (15, 19, 23). The perioperative complications of CRT-D and CRT-P devices in NICM patients were not contrasted in any of the studies. In the final analysis, we included 11 studies with a total of 28,768 CRT-eligible patients. There were 16,788 CRT-D implantations and 11,980 CRT-P implantations. Drozd et al. (17) conducted a prospective cohort study, whereas the other cohort studies included were retrospective. Table 1 summarizes the baseline characteristics of all included studies.

**Table 1:** Baseline characteristics of the included studies in this meta-analysis.

Name	Single vs. multicenter/country	Study design	Randomized vs cohort	Publication	Year	Nonischemic CRT-D patients	Nonischemic CRT-P patients	Follow-up time (month)	Outcomes	Adjustments made
Kutyifa et al.	Single-center/Hungary	Retrospective	Cohort	Eur Heart J HF	2014	209	458	28 (median)	All-cause mortality, LVEF improvement	Age and sex
Witt et al.	Single-center/Denmark	Retrospective	Cohort	EP Europace	2016	122	305	48 (median)	All-cause mortality	Apriori confounders
Barra et al.	Multicenter/European consortium	Retrospective	Cohort	JACC	2017	1,943	682	41.4 (median)	All-cause mortality	Propensity score
Drozd et al.	Single-center/United Kingdom	Prospective	Cohort	Jour Cardiovas Med	2017	25	240	35.2 (median)	All-cause mortality	Propensity score
Leyva et al.	Multicenter/United Kingdom	Retrospective	Cohort	EP Europace	2018	114	116	56.4 (median)	All-cause mortality, HF hospitalization, MACE, cardiac mortality, death from pump failure, SCD or hospitalization of VT/VF	Propensity score
Wang et al.	Single-center/United States	Retrospective	Cohort	Indian Pacing	2019	93	42	46 (median)	All-cause mortality	LVEF, ACE inhibitor use, Charlson comorbidity index
Saba et al.	Multicenter/US Medicare database	Retrospective	Cohort	Heart Rhythm	2019	4,359	1,236	60 (median)	All-cause mortality, time to first cardiac, medical costs	Cox proportional hazards
Køber et al.	Multicenter, randomized/Danish	RCT	RCT	N. Engl. J. Med.	2016	322	323	67.6 (median)	All-cause mortality, Death from Any Cause, Serious device infection	Time-to-event methods
Gras et al.	Multicenter/French	Retrospective	Cohort	Europace	2020	9,129	8,176	30.4 (median)	All-cause mortality; Cardiovascular death; Noncardiovascular death	Propensity score
Liang et al.	Single-center/China	Retrospective	Cohort	J CARDIOL	2020	202	117	36 (median)	All-cause Mortality	Propensity score
Doran et al.	Multicenter/United States	RCT	RCT	JACC Heart Fail	2021	270	285	17.1 (median)	All-cause Mortality; Sudden Cardiac Death; Cardiovascular mortality or Heart failure hospitalization	Cox proportional hazards models

MONICA, Monitoring of Trends and Determinants in Cardiovascular Disease Augsburg; MONICA, Monitoring trends and determinants on cardiovascular diseases; NHIC, National Health Insurance Corporation; NOS, The Newcastle‒Ottawa Scale items.

ACE, inhibitor indicates angiotensin-converting enzyme inhibitor; CRT, cardiac resynchronization therapy; CRT-D, CRT defibrillator; CRT-P, CRT pacemaker; HF, heart failure; LVEF, left ventricular ejection fraction; MACE, major adverse cardiac event; SCD, sudden cardiac death; VF, ventricular fibrillation; VT, ventricular tachycardia; RCT, randomized controlled trial.

We assessed the included randomized controlled trials using the Cochrane Risk of Bias Instrument criteria. The risk of bias with Kber et al. and Doran et al. was thought to be low overall (Supplementary Table S2 in the Data Supplement). The NOS risk of bias tool was used to evaluate the level of the literature of the other nine studies, and the overall quality of the literature was deemed moderate (Supplementary Table S3 in the Data Supplement). Each study considered confounding factors, used relevant methods to adjust for confounding factors, and provided adjusted HRs.

### All-cause mortality

**Figure 2:**
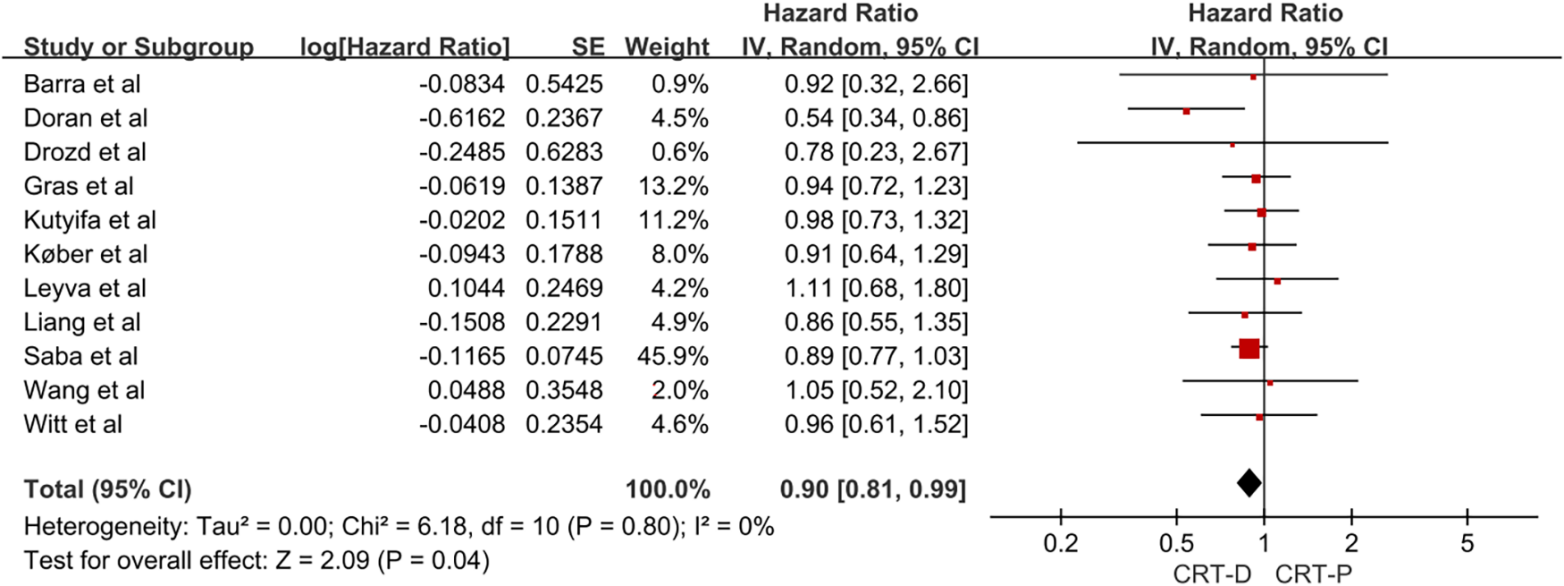
Forest plot for the risk of all-cause mortality in NICM heart failure patients. CI, confidence interval; SE, standard error; IV, inverse of the variance.

We conducted a pooled analysis of 11 studies, and the results using a random-effects model revealed that the adjusted HR for all-cause mortality in patients with NICM combined with HF treated with CRT-D vs. CRT-P was 0.90 (95% CI, 0.81–0.99; Figure 2). The 11 studies had no significant heterogeneity (I^2 ^= 0%). In the study of all-cause mortality, the funnel plot revealed no significant asymmetry (Supplementary Figure S1 in the Data Supplement).

**Figure 3:**
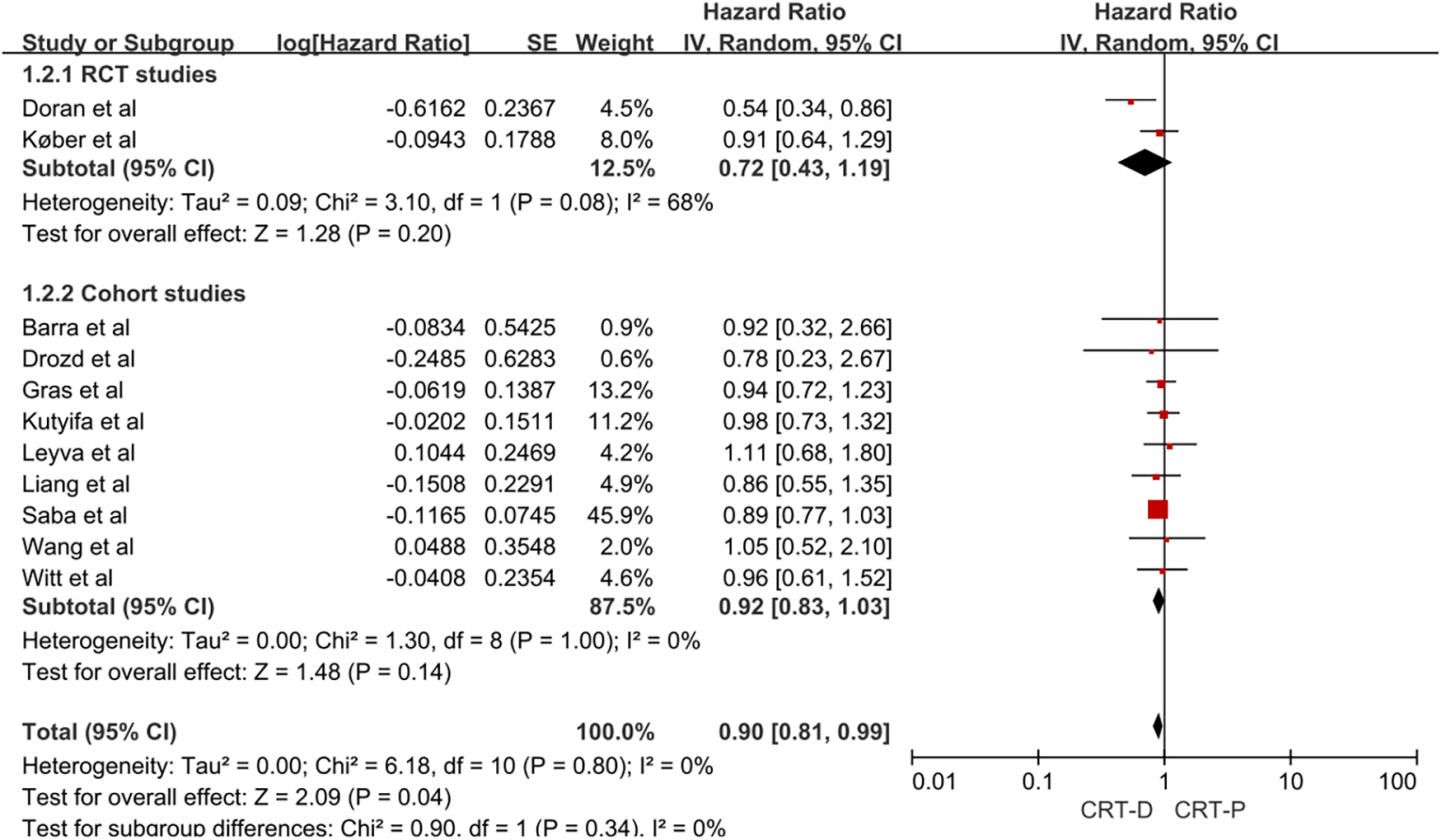
Forest plot for subgroup analysis of all-cause mortality risk in patients with NICM heart failure. CI, confidence interval; SE, standard error; IV, inverse of the variance.

Given the methodological differences between RCT and cohort studies, as well as the possibility of bias in the results, we conducted a subgroup analysis of the two types of research. In a meta-analysis of two RCTs, the adjusted HR of all-cause mortality for CRT-D vs. CRT-P in patients with NICM and HF was 0.72 (95% CI, 0.43–1.19; Figure 3). In a meta-analysis of nine cohort studies, the adjusted HR of all-cause mortality for CRT-D vs. CRT-P in patients with NICM and HF was 0.92 (95% CI, 0.83–1.03; Figure 3).

### Infection

Only one review (7) investigated the risk of infection and discovered no difference between CRT-D and CRT-P patients [HR, 0.82 (0.29–2.20)].

### Sudden death and cardiac mortality

Two cohort studies (19, 23) and 1 RCT study (15) reported sudden cardiac death and cardiac mortality.

Doran et al. (15) found that implantation of CRT-D reduced the risk of sudden cardiac death in non-ischemic cardiomyopathy compared to CRT-P [HR, 0.29 (0.11–0.78)]. The pooled analysis of Leyva et al. (19) and Gras et al. (23) found no difference in the risk of cardiac mortality in patients receiving CRT-D vs. CRT-P [HR, 0.89 (0.66–1.20)].

## Discussion

In the total combined analysis of this systematic review and meta-analysis, we found a slight reduction in the risk of all-cause mortality with CRT-D vs. CRT-P in patients with NICM, This finding suggests that in patients with NICM, CRT-D would be more beneficial to CRT-P. But no such difference was found in the subgroup analysis of the RCTs and cohort studies.

### Exploration of differences in the results of RCTs

It is worth noting that we found only two *post hoc* analyses of RCTs (7, 15). The results of the RCT were completely diverse. In NICM patients eligible for CRT indications, the DANISH trial (25) found no difference in the risk of all-cause mortality with CRT-D vs. CRT-P. This RCT, however, was not designed to determine whether additional defibrillation would benefit NICM patients eligible for CRT indications, and these data are only a subset of the data from this RCT. The COMPANION trial was conducted earlier than the DANISH trial, and the risk of all-cause mortality was significantly lower in patients with NICM in CRT-D vs. CRT-P (26). Differences in study design and participant clinical baseline characteristics between the DANISH and COMPANION trials could explain differences in the risk of all-cause mortality in patients with NICM. First, COMPANION trial patients had higher graded severity of cardiac function, more severe structural and functional left ventricle remodeling, and lower LVEF than DANISH trial patients. COMPANION, unlike DANISH, only randomized patients with HFrEF in functional class III or IV. Second, in the COMPANION trial, the mean LVEF in NICM patients was 20%, compared to 25% in DANISH NICM patients. Furthermore, the COMPANION trial’s inclusion criteria included left ventricular dilatation and a delayed QRS time frame, with two-thirds of the participants having left bundle branch block (27). Third, the duration of the disease could have influenced the outcome. Another significant difference between the two studies is that the DANISH study had a 5-year follow-up period, whereas the COMPANION study only had 1.4 years. All of these important factors would cause the results of the two trials to differ. In the COMPANION study, the annual mortality rate for NICM patients was 11.2%, while the annual mortality rate for NICM patients in the DANISH study was 4.0%. In conclusion, the difference in ICD benefit between the DANISH and COMPANION trials in NICM patients eligible for CRT indications was caused by differences in the severity of HFrEF, and thus, the risk of all-cause mortality was higher in COMPANION NICM patients. The current two RCTs have the following limitations. First, neither RCT was designed specifically to compare CRT-D vs. CRT-P prognosis in NICM patients but instead was a subgroup analysis of ICD therapy. Second, due to the small number of NICM patients included in the study, further subgroup analysis was not possible. Third, both studies were conducted prior to the regulatory approval of sodium-glucose cotransporter-2 inhibitors and sacubitril/valsartan, and more research is needed to compare the effects of CRT-D vs. CRT-P in NICM patients in the context of novel anti-cardiac failure drug therapy. In conclusion, high-quality RCTs comparing CRT-D vs. CRT-P prognosis in NICM patients are required in the future.

### Exploration of differences in the results of cohort studies

The pooled analysis of RCTs was not designed to determine whether adding defibrillation to CRT-eligible patients with NICM would benefit them. As a result, we included nine cohort studies with a total of 27,568 NICM patients, and after controlling for confounding factors such as baseline variables in NICM patients eligible for CRT indications, there was no statistically significant difference in all-cause mortality between CRT-D and CRT-P. In contrast to Patel et al’s systematic review and meta-analysis (12), in which we included more cohort studies, it is worth noting that CRT appears to reduce the risk of all-cause mortality in NICM patients compared to CRT-D, albeit by a statistically insignificant margin due to the small number of cohort studies. More cohort studies are required in the future to evaluate this difference.

### Impact on clinical practice

It is still debated whether additional defibrillator leads should be implanted in patients with NICM and heart failure who are candidates for CRT. There is no denying that the ability of implanted defibrillators to terminate life-threatening malignant ventricular arrhythmias is a critical benefit. However, REVERSE trial data revealed that patients implanted with a CRT-D device cost approximately $200,000 more than those implanted with a CRT-P device and had a battery life of only 3.6 years (28). According to previous research, the median 12-month cost for patients receiving CRT-D was $64,891 vs. $5,220 for patients receiving CRT-P (20). Patients frequently have difficulty tolerating defibrillation-related inappropriate electrical shocks from implanted defibrillator leads. Electrophysiologists may be hesitant to implant defibrillation leads in patients with specific types of NICM, particularly because some patients, such as those with pacing-induced cardiomyopathy or LBBB-induced cardiomyopathy, may benefit more from CRT therapy.

As a primary recommendation, the 2017 AHA/ACC/HRS Guidelines for the Treatment of Patients with Ventricular Arrhythmias and the Prevention of Sudden Cardiac Death suggest that NICM patients with an LVEF ≤35% after three months of drug therapy have a defibrillator implanted (29). As a result, our findings highlight the importance of adequately powered RCTs in answering this question definitively. Despite the fact that current RCT studies have limitations, they do not adequately answer whether CRT-D is more beneficial than CRT-P in NICM patients. Despite the low level of evidence, observational studies can provide preliminary information on whether CRT-D is beneficial in NICM patients with CRT indications. Given the serious risk of bias in observational studies, only rigorous RCTs can compensate for these shortcomings. Unfortunately, neither the RCTs nor the observational studies found a statistically significant difference in the risk of all-cause mortality in NICM patients treated with CRT-D vs. CRT-P. As a result, electrophysiologists should use individualized treatment to determine which NICM patients are more likely to benefit from CRT-D treatment until more RCTs can definitively answer this question.

### Limitations

The current study has some limitations that should be mentioned. First, there were very few RCT studies included, and the risk of death in NICM patients receiving CRT-D vs. CRT-P was only a subgroup analysis among RCT studies. Second, there were fewer prospective cohort studies included. Despite the fact that all of the observational studies we included controlled for baseline covariates, the effect of residual confounding factors such as age or other comorbidities may influence whether defibrillators improve the risk of survival in NICM. Because of the inherent limitations of observational studies, the level of evidence that can be provided is low. Third, due to limited data, we were unable to perform a pooled analysis of secondary outcomes. Although different secondary outcomes were considered and could be inferred in the search strategy designed, such as the risk of hospitalization, infection, and cardiovascular death, we were unable to perform a pooled analysis of secondary outcomes.

## Conclusion

With the addition of defibrillation leads, we found a significantly lower risk of all-cause mortality in patients with NICM, but this association was not found in subgroup analyses of RCTs and observational studies. More randomized trials are required to help inform decisions about the best device for this patient group.
