# Supplementary material for: An updated meta-analysis of cardiac resynchronization therapy with or without defibrillation in patients with nonischemic cardiomyopathy
Source: Front Cardiovasc Med. 2023 Jul 12;10:1078570. doi: 10.3389/fcvm.2023.1078570 (PMC10370697; doi:10.3389/fcvm.2023.1078570)

**Data Supplement**

**Supplementary Table 1. The search strategies of this meta-analysis**

|  | **Search terms** | **No.** |
| --- | --- | --- |
| **PubMed** | | |
| #1 | cardiomyopathies[Title/Abstract] | 6,853 |
| #2 | cardiomyopathy[Title/Abstract] | 80,361 |
| #3 | CRT[Title/Abstract] | 19,069 |
| #4 | "Cardiac Resynchronization Therapy"[Mesh] | 8,162 |
| #5 | #1 OR #2 | 83,782 |
| #6 | #3 OR #4 | 22,083 |
| #8 | #5 AND #6 | 1,439 |
| **Embase** | | |
| #1 | cardiomyopathies | 9,405 |
| #2 | cardiomyopathy | 125,210 |
| #3 | CRT | 39,888 |
| #4 | Cardiac Resynchronization Therapy | 14,186 |
| #5 | #1 OR #2 | 130,072 |
| #6 | #3 OR #4 | 43,548 |
| #7 | #5 AND #6 | 3,666 |
| **cochrane** | | |
| #1 | cardiomyopathies | 4,776 |
| #2 | cardiomyopathy | 4,780 |
| #3 | CRT | 4,977 |
| #4 | Cardiac Resynchronization Therapy | 445 |
| #5 | #1 OR #2 | 4,780 |
| #6 | #3 OR #4 | 5,060 |
| #7 | #5 AND #6 | 247 |

**Supplementary Table 2. Risk of bias assessment for RCTs and post-hoc analyses of RCTs**

|  | Random sequence generation (**selection bias**) | Allocation concealment (**selection bias**) | Blinding of participants and personnel (**performance bias**) | Blinding of outcome assessment (**detection bias**) | Incomplete  outcome data  (**attrition**  **bias**) | Selective reporting (**reporting bias**) | **Other bias** | **Total*** |
| --- | --- | --- | --- | --- | --- | --- | --- | --- |
| Køber et al | Moderate | Low | Low | Low | Low | Low | Low | Low |
| Doran et al | Low | Low | Moderate | Low | Low | Low | Low | Low |

*We defined as “low risk” when 3 out of 5 biases were “low”.

**Supplementary Table 3. Quality assessment for the included observational studies**

| Included studies | Selection (0-4 points) | | | | Comparability (0-2 points) | | Outcome (0-3 points) | | | Total poitns* |
| --- | --- | --- | --- | --- | --- | --- | --- | --- | --- | --- |
|  | Representativeness of Exposed Cohort | Selection of Non-Exposed Cohort | Ascertainment of Exposure | Demonstration That Outcome of Interest Was Not Present at Start of Study | Adjust for the important Risk factors | Adjust for other risk factors | Assessment of outcome | Follow-up length | Loss to follow-up rate |  |
| Kutyifa et al | * | * | * | * | * |  | * |  |  | 6 |
| Witt et al | * | * | * | * | * | * | * |  |  | 7 |
| Barra et al | * | * | * | * | * | * | * | * |  | 8 |
| Drozd et al | * | * | * | * | * |  | * |  |  | 6 |
| Leyva et al | * | * | * | * | * | * | * |  |  | 7 |
| Wang et al | * | * | * | * | * |  | * |  |  | 6 |
| Saba et al | * | * | * | * | * | * | * |  |  | 7 |
| Gras et al | * | * | * | * | * | * | * |  |  | 7 |
| Liang et al | * | * | * | * | * | * | * | * |  | 8 |

＊The Newcastle-Ottawa Scale (NOS) items, with a total score of 9 points, were used to evaluate the quality of the post-hoc analyses of RCTs and observational study which involve the selection of cohorts (0-4 points), the comparability of cohorts (0-2 points), and the assessment of the outcome (0-3 points)

**Supplementary Figure 1.The funnel plots for for all-cause mortality**


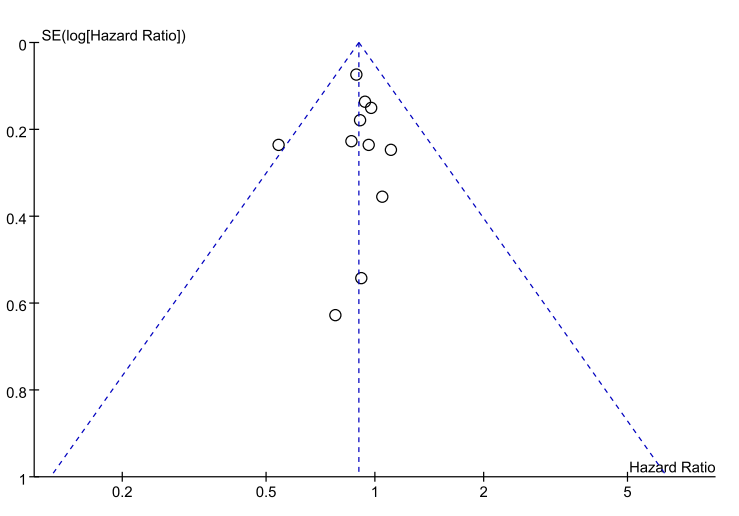

Supplement: Supplementary file 1 [file Datasheet1.docx]
